# Evidence for synergistic effects of *PRNP* and *ATP7B* mutations in severe neuropsychiatric deterioration

**DOI:** 10.1186/1471-2350-15-22

**Published:** 2014-02-20

**Authors:** Nauzer Forbes, Susan Goodwin, Kevin Woodward, David G Morgan, Lauren Brady, Michael B Coulthart, Mark A Tarnopolsky

**Affiliations:** 1 Department of Medicine, McMaster University, Hamilton, Canada; 2 Department of Pediatrics, McMaster University, Hamilton, Canada; 3 National Microbiology Laboratory, Public Health Agency of Canada, Winnipeg, Canada; 4 Neuromuscular and Neurometabolic Disease, Department of Pediatrics, McMaster University, 1200 Main St. W., HSC-2H26, Hamilton, ON L8N 3Z5, Canada

**Keywords:** Wilson’s disease, Prion disease, Copper, *ATP7B*, *PRNP*, Prion protein, PrP, Synergistic mutations, Movement disorder

## Abstract

**Background:**

Wilson’s disease (WD), a rare cause of neuropsychiatric deterioration, is associated with mutations in the *ATP7B* gene. Prion diseases are also rare causes of neuropsychiatric deterioration that can occur sporadically without an identifiable cause, or can be attributed to mutations in the *PRNP* gene.

**Case presentation:**

Here we describe a biological “experiment of nature” in which a patient presented with severe neuropsychiatric decline and strong biochemical evidence of WD. Genetic analysis revealed that he was a compound heterozygote for two *ATP7B* sequence variants (c.2165dupT, p.Arg723Glufs*32; and c.4039G > A, p.Gly1347Ser), the first having been reported once previously, and the second being novel. In addition, the patient was heterozygous for a *PRNP* variant, c.160G > A, p.Gly54Ser, that has been reported in a neuropsychiatric patient only once previously in association with a similarly severe clinical course of neuropsychiatric disease and early age of onset, but no accompanying information on *ATP7B* genotype. Of particular interest was the observation that the patient’s older sister, who carried the same *ATP7B* genotype and laboratory evidence for biochemical WD but was clinically asymptomatic, lacked the *PRNP* variant allele.

**Conclusions:**

We propose that synergism may occur between at least some allelic variants of *ATP7B* and *PRNP*, possibly exerted through effects on cellular copper metabolism.

## Background

Wilson’s disease (WD, OMIM #277900) is an autosomal recessive genetic disorder of copper metabolism [1], with an estimated global prevalence as high as 1 in 30,000 [2], implying a Hardy-Weinberg allele frequency of approximately 0.6% (carrier frequency of about 1 in 90). The implicated gene in WD is *ATP7B*, which encodes a copper-transporting P-type ATPase, ATP7B, and is mainly expressed in hepatic, renal, ocular and neural tissues. More than 500 *ATP7B* variant alleles have been described since its discovery in 1993 (http://www.wilsondisease.med.ualberta.ca/database.asp) [3,4]. The primary pathophysiologic effect of these genotypes is multi-organ deposition of copper with mainly hepatic, neurologic and psychiatric clinical manifestations. Forty to fifty percent of WD patients have hepatic symptoms as the initial manifestation, although neurologic and psychiatric presentations are also relatively common, and about 50% of all patients will have neuropsychiatric manifestations during their disease course, including movement disorders (bradykinesia, rigidity, tremor, dystonia), ataxia, cognitive impairment, depression and psychosis. The classical approach to diagnosis of WD relies upon recognition of neurological and/or hepatic features, finding of Kayser-Fleischer rings (~ 2/3 of those with neurological disease), and measurement of altered biochemical markers in the serum (low copper and ceruloplasmin) and/or urine (high 24 h copper excretion) [5]. However, with increasing understanding of the structure, function and mutational spectrum of *ATP7B* and ATP7B, and declining costs for DNA analysis, genetic testing is increasingly used for definitive differential diagnosis of the disease [6].

Prion diseases (OMIM #123400, #137440, #600072) also result in progressive, irreversible neurodegeneration, with cognitive and psychiatric manifestations that can overlap significantly with the phenotype of WD. Although clinical presentation of prion disease (including genetic forms) is highly varied, neurological and/or psychiatric manifestations are a consistent feature in nearly all cases. Human prion diseases usually occur sporadically, or very rarely through infectious transmission, but can also arise (in 5–15% of cases) in association with phenotypically dominant mutations in the autosomal gene (*PRNP*) that encodes the prion glycoprotein PrP [7,8]. Etiologically, prion diseases are classified as proteopathies, with pathobiology triggered by post-translational conversion of the normal conformation of PrP (PrP^C^) into a misfolded isoform (PrP^Sc^) that is deposited as insoluble aggregates particularly in neural tissues(Prusiner, 1998). Although the normal physiological functions of PrP^C^ are less well understood than those of ATP7B, evidence is accumulating to support the hypothesis that these functions are related to copper, particularly in the brain [9-11].

Here we report the finding of nonsynonymous coding-sequence variants in both *ATP7B* and *PRNP* genes in a patient with rapidly progressive neurological WD. We postulate that synergistic interaction between the observed *ATP7B* and *PRNP* variants, mediated by effects of their encoded variant proteins on copper metabolism, may underlie the patient’s severe course of neurologic deterioration.

## Case presentation

A 34-year-old man from the Punjab region of India, who had immigrated to Canada in 2010, having developed a gradual onset of a bilateral intention tremor, worsening over the course of 7 years. This was initially diagnosed as spinocerebellar ataxia. Over the previous 2 years he had experienced various progressive psychiatric disturbances including severe depression, anxiety and mood lability. This constellation of symptoms had been variously diagnosed as depression, bipolar disorder and bipolar affective disorder, for which trials of several mood stabilizers, antidepressants and antipsychotics had been attempted with limited, modest, short-term benefits.

In the year following emigration, the patient experienced a rapid decline in overall function, marked by an incident involving a trip and a fall. He began experiencing gait instability and imbalance, rigidity, scanning speech, dysphagia, and eventually choking episodes. The neuropsychiatric symptoms worsened over several months as the patient concomitantly experienced restlessness, sexual disinhibition, aggression and social inappropriateness. By the summer of 2011, his balance and rigidity had further deteriorated preventing him from ambulating, and thus prompting his family to seek further medical assessment. His symptoms culminated in an in-hospital episode of acute dystonia in July of 2011, prompting admission. At the time of admission following this episode, the patient was on lithium, quetiapine, propranolol, and zopiclone.

The patient’s family history revealed no known consanguinity and the only significant neurological disorder was in the now deceased proband’s maternal grandfather who developed a rapidly progressive gait disturbance and cognitive impairment at age 62 y and died ~ 8 months after the onset with a first generation CT scan showing “cerebral atrophy”. Neurological examinations of the proband’s mother (age 61 y), father (age 62 y) and older sister (age 39 y) were normal, aside from a mild intention tremor in the mother. Physical examination revealed normal vital signs but the patient was agitated, with labile mood and sporadic emotional outbursts. Detailed neurologic examination revealed the presence of scanning dysarthria, severe rigidity in both upper and lower extremities, hyperreflexia in arms and legs, appendicular dystonia, dysdiadochokinesia and truncal ataxia. Ophthalmologic examination did not reveal Kayser-Fleischer rings by fundoscopy; however, the patient was unable to be positioned for a slit-lamp examination because of severe rigidity and tremor. Cardiac, respiratory and abdominal examinations were unremarkable, with no evidence of hepatosplenomegaly or ascites. There were no stigmata of acute or chronic liver disease, and no asterixis.

Brain MRI revealed generalized atrophy in addition to “giant panda sign” at the level of the midbrain, consistent with Wilson’s disease. Abdominal ultrasound showed coarse liver echogenicity and irregular contours with a height of 10.2 cm in the mid-clavicular line, suggestive of cirrhosis. Liver biopsy confirmed grade 2–3 fibrosis and bridging, with Orcein stain negative for copper deposition (no direct biochemical measurement). A complete biochemical and pathologic workup was negative for viral hepatitis, alpha-1 anti-trypsin disease, hemochromatosis, and autoimmune hepatitis. Echocardiogram showed normal ejection fraction with no hypertrophy or abnormal deposition.

Routine biochemical investigations of blood and urine were within normal limits, including a complete blood count, extended electrolyte panel and tests of renal function. Liver enzymes were normal, as were tests of synthetic liver function. Tests of copper metabolism showed a markedly low serum ceruloplasmin of 0.05 (normal, > 0.21 g/L), low serum copper of 3.4 (normal, > 11 μmol/L), and high 24-hour urine copper of 1.8 (normal, < 0.6 μmol/24 h).

The patient was then treated with standard chelating therapy and zinc supplementation. Initially, he was started on penicillamine, but experienced commonly described adverse effects of severe neurologic deterioration and fever, warranting a change to trientene, which he is still taking along with zinc. His 24-hour urine copper value rose ten-fold to 17.7 μmol after 3 weeks of chelation. The patient’s psychiatric issues largely resolved but he did remain on the aforementioned psychotropic medications. He has made small gains in terms of recovery of neurologic function, but remains mute, wheelchair bound and dystonic, in spite of modest cognitive recovery when assessed one year after initiation of treatment.

Based upon the patient’s history of a severely progressive neurological disorder with basal-ganglia symptoms/signs, ataxia and a major antecedent psychiatric presentation, diagnoses of either WD or genetic prion disease were considered, and DNA sequence analysis of *ATP7B* and *PRNP* was requested. Analysis of the *ATP7B* gene was performed by the University of Chicago Genetic Services Laboratory in Chicago, Illinois through Sanger sequencing of all coding exons and intron/exon boundaries (reference sequence: GenBank NM_000053, transcript variant 1). Sanger sequencing of the single coding exon (exon 2) of the *PRNP* gene was performed by the Canadian Creutzfeldt-Jakob Disease Surveillance System, Public Health Agency of Canada (reference sequence: GenBank M13899).

DNA sequencing revealed that the patient was a compound heterozygote for two different *ATP7B* sequence variants (c.2165dupT; c.4039G > A). His *PRNP* gene lacked known pathogenic sequence variants, but he was heterozygous for a non-synonymous sequence variant (c.160G > A, p.Gly54Ser), as well as homozygous (ATG/ATG) for a common, non-pathogenic single-nucleotide polymorphism (c.385A > G, p.Met129Val) at codon 129.

**Figure 1:**
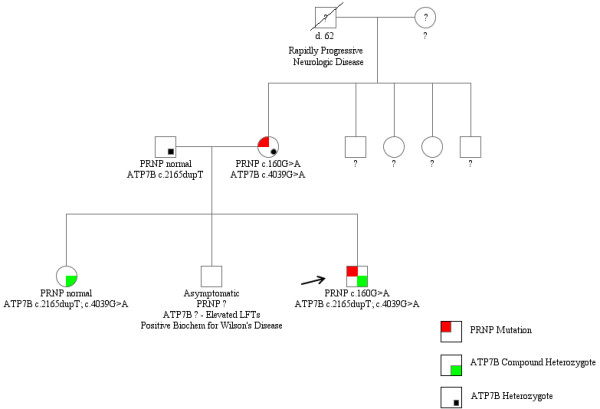
**Pedigree showing *ATP7B *and *PRNP *mutations among the proband and family members.** Pedigree was created using the Progeny Free Online Pedigree Tool (http://www.progenygenetics.com/).

A family study (Figure 1) revealed that the parents of the proband were each carriers of one of the two different *ATP7B* mutations (c.2165dupT in the father, and c.4039G > A in the mother). His mother carried the *PRNP* c.160G > A variant and was heterozygous ATG/GTG (Met/Val) at codon 129, while his father did not carry the *PRNP* c.160G > A variant and was homozygous ATG/ATG (Met + Met) at codon 129. The sister of the proband was found to be a compound heterozygote for the same two *ATP7B* variants carried by her brother, while lacking the c.160G > A variant and being heterozygous ATG/GTG (Met + Val) at codon 129 of *PRNP*. However, despite exhibiting a classical biochemical phenotype for Wilson’s disease with a low serum copper level of 2.1 μmol/L, a low ceruloplasmin of 0.12 g/L, and a high 24 h urine copper level of 1.1 μmol (normal values as above), she was asymptomatic, with a normal MRI and neurological examination.

## Conclusions

We describe a case of severe, rapidly progressive neurological WD in a patient who was found to carry heterozygous allelic variants in the two genes (*ATP7B* and *PRNP*) that have been linked to WD and prion disease, respectively. We consider it remarkable that the patient’s sister did not display a WD clinical phenotype (and normal MRI), in spite of having the same *ATP7B* genotype and biochemical abnormalities. Without confirmation of the genotype of the deceased maternal grandfather it is speculative as to whether or not he had the maternal genotype (*PRNP* c.160G > A; *ATP7B* c.4039G > A) that explained the later onset of a severe phenotypically similar neurological decline.

These findings may be interpreted to argue for rather impressive heterogeneity of genetic penetrance among siblings with identical *ATP7B* genotypes, conceivably mediated in part by genetic differences between the siblings at other, unexamined genetic loci. However, it is of interest that both ATP7B and PrP proteins have been biologically linked to cellular systems for copper handling and regulation in the brain [9,12]. Thus, despite the acknowledged limitations of our study based on its small size, we feel that the current findings, including both the severity of the patient’s disease and the phenotypic discordance between himself and his sibling, merit raising the question of possible synergistic interaction between the biochemical and/or cellular effects of the proteins encoded by the variant alleles we observed in these two genes, with an unknown mechanism but perhaps mediated by effects on copper metabolism. Other reports have linked variation in the progress of Wilson’s disease with the common p.Met129Val polymorphism in *PRNP*[13,14]. However, as discussed further below, the p.Gly54Ser *PRNP* variant we identified in this family is located in a different domain of the PrP protein that is directly implicated in copper metabolism.

The two cation-transporting P-type ATPases, ATP7A and ATP7B, are vital in both the incorporation of copper into the plasma protein ceruloplasmin and the biliary excretion of copper [12]. Structurally, the N-terminal region of these enzymes contains 6 heavy-metal-associated domains that each span approximately 70 amino acid residues and are involved in binding copper directly [15]. The C-terminal region contains 8 transmembrane (TM) domains, an E1-E2 ATPase domain, and a haloacid dehydrogenase domain that contains the catalytic active site. One of the two *ATP7B* variants observed in our patient, c.2165dupT in exon 8, results in a frame-shifted coding sequence, and is predicted to encode a truncated polypeptide with the heterologous sequence beginning with an Arg723Glu substitution within the TM2-TM3 cytosolic loop (Ala718–His724) and ending 32 amino acids downstream with a premature stop codon. This variant has been reported once previously in the literature, and is recorded twice in the Wilson disease mutation database [4,16]. The other variant, c.4039G > A in exon 20, leads to a Gly → Ser substitution at the evolutionarily conserved Gly1347 residue within the non-cytosolic TM7-TM8 loop (Gly1341–Gln1351). This variant has not been reported previously but is predicted to “affect protein function” by SIFT BLink (score = 0.00) and to be “probably damaging” by PolyPhen-2 (score = 1.00)(NP_000044.2). Thus, both of the encoded variant ATP7B proteins are predicted to be functionally hypomorphic, accounting for the proband and his sister’s low levels of serum copper and ceruloplasmin as well as elevated level of urine copper. Each of the variants independently affected copper metabolism to an expected lesser degree for each of the parents (mother = c.4039G > A; father = c.2165dupT), showed below normal concentrations of both serum copper and ceruloplasmin.

Although the biochemical and cellular functions of PrP^C^ are not yet as well-described as those of ATP7A and ATP7B, the N-terminal half of this protein is flexibly unstructured and probably multifunctional [17], and contains a domain of contiguous amino acid sequence repeat units, highly conserved among mammals [18]. In humans this domain, which spans PrP residues Pro51–Gln91, consists of 5 repeat units including an N-terminal nonapeptide lacking histidine (Pro-Gln-Gly-Gly-Gly-Trp-Gly-Gln) and 4 tandem copies of a His-containing octapeptide (Pro-His-Gly-Gly-Gly-Trp-Gly-Gln). The 4 octapeptides have been found to bind copper cooperatively with a physiologically relevant range of affinities, through coordination at the His residues as well as two deprotonated backbone amide nitrogens and a carbonyl oxygen of the following two Gly residues [9,19,20].

Physiologically, evidence is strong that PrP^C^ functions as a cuproprotein, potentially playing one or more roles as an antioxidant, chaperone, transporter, enzyme, or sensor, particularly at the neuronal synapse [9-11]. *PRNP* variant alleles with insertions [21], or deletions [22], of octapeptide repeat units result in prion disease in humans. For insertion alleles, disease phenotype (particularly age of onset) appears to be correlated with the number of extra octapeptide repeats; these phenotypic effects have been proposed to be mediated in turn by effects on copper binding behavior of the repeats [23]. In addition, experimental animal models of prion disease have been shown to be sensitive to either reduced or elevated levels of copper [24,25]. However, the specific role(s) of PrP^C^ in the copper-handling machinery of the brain remain incompletely understood.

The c.160G > A variant *PRNP* allele observed in our patient results in a predicted substitution of Ser for the second Gly within the nonapeptide unit of the PrP octapeptide repeat domain. In contrast with the 4 octapeptide units, the nonapeptide unit does not contain His, and no specific function has yet been proposed for it, including involvement in the binding of copper. Thus, at this time we cannot predict the precise biochemical or physiological effects of the c.160G > A variant. However, given its high sequence similarity and position immediately adjacent to the octapeptide-repeat array, as well as the observation that the presence, position and sequence of the nonapeptide unit are highly conserved among mammalian species [18], it is worth considering the hypothesis that the Gly54Ser substitution subtly alters the steric interaction of PrP^C^ with copper, and that this altered interaction promotes the neuropathogenic effects of elevated brain copper levels in WD.

In the sole other published report describing a case of neuropsychiatric disease in an individual with the same *PRNP* genotype as our proband (c.160G > A; codon 129 ATG/ATG), a 43-year-old female Ugandan patient of similar ethnogeographic background (South East Asian) presented with dysarthria, which progressed into non-fluent dysphasia, progressive apraxia and extrapyramidal signs [26]. Five years after disease onset, she was mute and incapable of ambulation, with death following some months thereafter. At autopsy, widespread deposits of PrP^Sc^ were observed with immunohistochemical analysis of cerebral and cerebellar tissue, in a pattern not previously observed in genetic prion diseases. Intriguingly, the c.160G > A variant was also found in a single healthy Gujarati individual screened at the time of initial patient referral, and at very low frequencies in reportedly healthy Pakistani, Chinese and Middle-Eastern groups included in the CEPH human genome diversity cell line panel. These findings strongly suggest that the c.160G > A *PRNP* variant is not sufficient in itself to cause prion disease, although the absence of evidence for such pathogenic effects from the healthy control populations does not completely exclude the possibility of lower-penetrance effects on prion disease risk. However, we agree with the authors of the above-mentioned study [26], that this patient’s disease presentation remains incompletely explained. It is tempting to suggest that abnormal copper metabolism might have contributed to her clinical disease, perhaps with the involvement of undocumented genetic variation at *ATP7B*.

In summary, we suggest that the simultaneous presence of protein-coding sequence variants in *ATP7B* and *PRNP* in a patient with neurological Wilson’s disease constitutes a serendipitous biological “experiment of nature” that will be of interest both to clinicians from a diagnostic perspective, and also to basic scientists interested in the (patho)physiologic functions of ATP7B and PrP, including potential biochemical or metabolic synergistic effects mediated by copper metabolism of the human brain.

## Consent

Written informed consent was obtained from the patient, his wife and family members for publication of this Case report and any accompanying images. A copy of the written consent is available for review by the Editor of this journal. Permission to publish case reports regarding new mutations was discussed with the Chairperson of the Hamilton Health Sciences Research Ethics Board and written consent was given for this purpose.

## Competing interests

None of the authors report any competing interests (financial or otherwise) with respect to the current manuscript.

## Authors’ contributions

1) have made substantial contributions to conception and design, or acquisition of data, or analysis and interpretation of data (MT, MC, NF); 2) have been involved in drafting the manuscript or revising it critically for important intellectual content (all authors); and 3) have given final approval of the version to be published (all authors).

## Pre-publication history

The pre-publication history for this paper can be accessed here:

http://www.biomedcentral.com/1471-2350/15/22/prepub
